# A cross-sectional study of point-of-care lactate testing in integrated community care management (ICCM) for children with acute respiratory illness in rural uganda

**DOI:** 10.1093/inthealth/ihae075

**Published:** 2024-11-09

**Authors:** Michael Matte, Natsumi Koyama, Dana Giandomenico, Emmanuel Baguma, Georget Kibaba, Moses Ntaro, Raquel Reyes, Edgar M Mulogo, Ross M Boyce, Emily J Ciccone

**Affiliations:** Department of Community Health, Mbarara University of Science & Technology, P.O. Box 1410 Mbarara, Uganda; Department of Epidemiology, University of North Carolina Gillings School of Global Public Health, Chapel Hill, NC; Institute for Global Health and Infectious Diseases, University of North Carolina School of Medicine, 130 Mason Farm Road, 2nd Floor, Chapel Hill, NC 27599, USA; Department of Community Health, Mbarara University of Science & Technology, P.O. Box 1410 Mbarara, Uganda; Department of Community Health, Mbarara University of Science & Technology, P.O. Box 1410 Mbarara, Uganda; Department of Community Health, Mbarara University of Science & Technology, P.O. Box 1410 Mbarara, Uganda; Division of Hospital Medicine, University of North Carolina School of Medicine, Chapel Hill, NC 27599, USA; Department of Community Health, Mbarara University of Science & Technology, P.O. Box 1410 Mbarara, Uganda; Department of Epidemiology, University of North Carolina Gillings School of Global Public Health, Chapel Hill, NC; Division of Infectious Diseases, University of North Carolina School of Medicine, 130 Mason Farm Road, 2nd floor, Chapel Hill, NC 27599, USA; Division of Infectious Diseases, University of North Carolina School of Medicine, 130 Mason Farm Road, 2nd floor, Chapel Hill, NC 27599, USA

**Keywords:** community health workers, integrated community case management, lactate, point-of-care testing

## Abstract

**Background:**

Integrated community case management (iCCM) programs leverage lay village health workers (VHWs) to carry out the initial evaluation of children with common conditions including malaria, pneumonia and diarrhea. Therefore, it is imperative that VHWs are able to identify children who are critically ill and require referral to a health facility. Elevated venous lactate levels have been associated with severe illness and adverse health outcomes, including death. However, lactic acidosis may not be recognized in rural settings because it is not routinely measured outside of hospitals and research studies. Point-of-care lactate tests may help identify patients in need of a higher level of care and improve VHWs’ ability to make timely and appropriate referrals.

**Methods:**

The study was a cross-sectional evaluation of children aged <5 y presenting to VHWs in rural southwestern Uganda with complaints of fever and cough. Demographics, clinical presentation, evaluation, management and disposition were recorded. VHWs were trained and instructed to perform lactate testing using a point-of-care assay in eligible participants.

**Results:**

During the study period, 238 children were enrolled and completed an initial assessment. Of the 204 participants included in the analysis, 113 (55.4%) were female, and the median (IQR) age was 23 (9–36) months. Most participants, 139/200 (69.5%), had negative results on the malaria rapid diagnostic test. The median lactate level was 2.1 mmol/L; 12% (24/204) had a lactate ≥3.5 mmol/L and only nine participants (4.4%) had a lactate ≥5 mmol/L. Having a lactate level above either cut-off was not associated with the presence of danger signs at presentation.

**Conclusions:**

Few children presenting with fever and cough to VHWs in western Uganda had elevated lactate levels. However, most of the children with elevated lactate levels did not otherwise satisfy established iCCM criteria based on physical examination findings for referral to a health facility. Therefore, while elevated lactate was not associated with danger signs in this small study, it is possible that there is under-recognition of severe illness using current iCCM guidelines.

## Introduction

Uganda, like many countries in sub-Saharan Africa (SSA), experiences a shortage of trained healthcare providers, falling far short of the WHO’s recommendation of 23 doctors, nurses and midwives per 10 000 population.^1^ These gaps are even more acute in rural areas, which bear a disproportionate burden of infectious diseases such as malaria.^2^ Furthermore, rugged terrain and limited transportation options may limit access to the formal public health system. In many areas, this has resulted in increased ‘task shifting’ of responsibilities from higher to lower level healthcare providers.^2^

To improve access to healthcare, in 2001, the government of Uganda created ‘Level 1’ health facilities, otherwise known as Village Health Teams (VHTs).^3^ Initially, these teams of community health workers were to serve as trainers for community-based health education and health-promotion campaigns. In 2010, the integrated community case management (iCCM) strategy was adopted to target the leading causes of morbidity and mortality in children aged <5 y: malaria, diarrheal disease and pneumonia.^4^

VHTs are comprised of lay community members who are selected by their respective villages and serve a 5-y term. While prior clinical training is not required, each village health worker (VHW) ideally should be able to read and write.^5^ After selection, each VHW completes an intensive, 5-d iCCM training course, typically conducted by the Ministry of Health (or a designated iCCM trainer of trainees previously trained by the Ministry of Health). During these sessions, VHWs learn how to diagnose and treat children with malaria, diarrhea and pneumonia with the help of the Sick Child Job Aid, a clinical decision-making algorithm.^4^,^6^ With greater implementation, VHWs have achieved positive health outcomes, and their work is well accepted within the community.^7–11^

VHWs are, however, only trained to treat uncomplicated malaria, diarrhea and pneumonia in children aged <5 y. Children presenting with specified ‘danger signs’, including unconsciousness, chest in-drawing and severe vomiting, are referred to a health facility for further evaluation and treatment. Yet, multiple studies have demonstrated that VHWs often do not appropriately refer eligible patients.^12^,^13^ This may be due to missed danger signs, some of which may be challenging to recognize, and lower adherence to referral guidelines for malaria rapid diagnostic test-negative patients due to diagnostic uncertainty. At the same time, many of those who are referred do not pursue further care.^8^,^14^,^15^ These systematic failures may partly explain why many deaths in rural SSA are not recorded in the formal health sector.^16^ This calls for objective, yet simple, tools that can be employed by VHWs to facilitate the timely identification and referral of children at a high risk of adverse clinical outcomes.

One biomarker that, when elevated, has been associated with severe illness and death is the venous lactate level.^17^,^18^ Elevated lactate was the most common manifestation of severe malaria in our previous study cohort,^19^ but may not be recognized because the physical signs of lactic acidosis are often subtle, particularly in children (e.g. rapid breathing). While traditional measurement of lactate within the confines of iCCM is not feasible as it is time-consuming and requires laboratory infrastructure, point-of-care lactate tests such as those that are used in sports medicine are affordable, available and have been previously shown to be accurate and highly predictive of severe disease in a hospital setting.^20^

A point-of-care lactate level may be particularly useful in the context of risk stratification for suspected pneumonia. In a cohort study conducted in Uganda from September 2013 to July 2015 involving 155 children admitted with WHO-defined clinical pneumonia, the in-hospital mortality rate was 14%. Admission lactate levels were strong predictors of mortality, outperforming several clinical risk scores, with higher levels correlating with increased mortality rates.^21^ Although lactate levels decreased over time in all children, they remained higher in those who died, underscoring the prognostic value of lactate measurement in low-resource settings.^21^

The objective of this study, therefore, was to preliminarily assess the potential role of point-of-care lactate testing as part of iCCM assessments and ultimately improve VHWs’ ability to make timely and appropriate referrals.

## Methods

### Study area/setting

**Figure 1.**
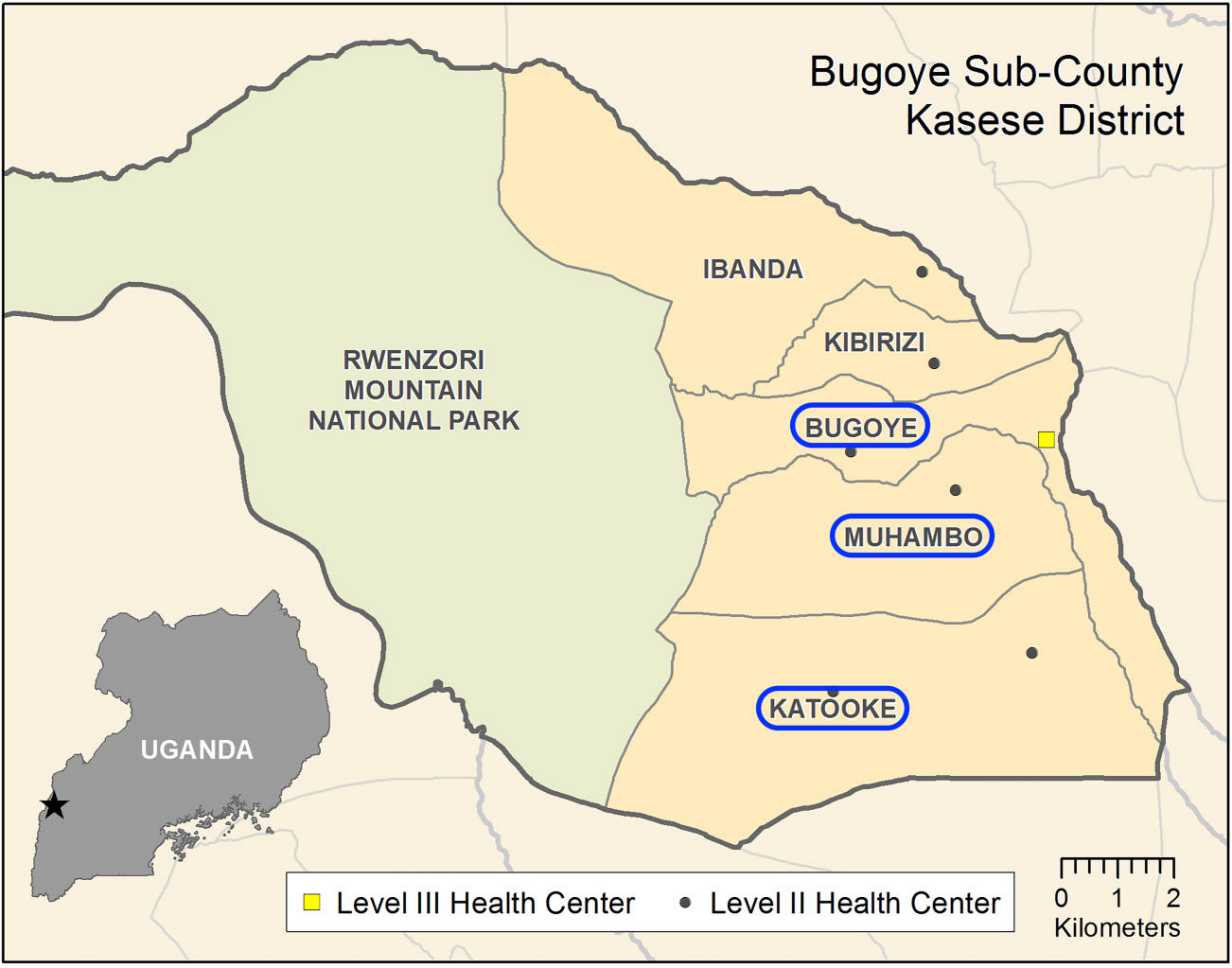
Map of the parishes in Bugoye subcounty from which the study population was recruited.

The study was conducted in the Bugoye subcounty of Kasese District in the Western Region of Uganda (Figure 1). The subcounty population is approximately 35 000 residents, nearly one-quarter of whom are children aged <5 y.^22^ The geography of the study area is highly varied. Most villages in the western part of the sub-county are characterized by deep river valleys and steep hillsides with elevations up to 2000 m. By contrast, the surroundings of those villages located to the east are defined by low-lying, level terrain. Therefore, this population is broadly representative of similar populations in malaria-endemic, rural areas with limited access to healthcare. In a recent retrospective review of the iCCM program in the subcounty, acute respiratory illnesses (ARI), specifically cough/fast breathing, were the second most common reason for VHW visit after fever, representing 45% of the 18 430 cases documented.^23^

### Study design

We conducted this study among children presenting to VHWs in the Bugoye, Muhambo and Katooke parishes of Bugoye subcounty in Kasese District during November 2019–February 2021 (Figure 1). It was initially designed as a prospective observational study, but due to a high proportion of missing follow-up assessments (70%), only cross-sectional data from the initial visit to the VHWs are reported.

### Eligibility and study procedures

Prior to study implementation, the five participating VHWs completed a 1-wk practical training course conducted by a senior laboratory technician of Bugoye Health Center III and the study staff covering human subjects research, use of the point-of-care lactate meter machine and data entry. The selected VHWs had previously completed the Ministry of Health-recommended iCCM training. Study staff also conducted spontaneous supervisory visits to reinforce adherence to protocol.

All children aged <5 y from Bugoye subcounty presenting to participating VHWs with symptoms of fever and cough were eligible. After the VHW read a standard form describing the study objectives, procedures and associated risks, the child's caregiver was asked to provide written consent. Children of caregivers electing not to participate underwent routine evaluation and treatment as per the local protocols.

For each participant, VHWs first completed a basic questionnaire documenting demographic and socioeconomic characteristics (Supplementary Material). The VHWs evaluated participating children according to standard iCCM protocols, assessing symptoms, measuring vital signs and performing a histidine-rich protein 2-based malaria rapid diagnostic test (mRDT). They also measured the venous lactate on capillary blood obtained via finger-prick using the Lactate Plus lactate meter (Nova Biomedical, Waltham, MA, USA). VHWs determined treatment and referral plans in accordance with the iCCM protocols if lactate levels were <5 mmol/L. VHWs were instructed to refer children with a lactate ≥5 mmol/L to the local health facility for further evaluation regardless of the algorithm after providing appropriate prereferral treatment. Treatment and disposition plans (i.e. discharge home vs referral to health center) were recorded.

At the end of each month, the VHWs submitted their monthly registers, consent forms and data collection forms to the health facility where the study team would crosscheck reports before entering the data into a secure REDCap database software (developed by Vanderbilt University in Nashville, Tennessee, USA).^24^

### Outcomes and definitions

The primary outcome of interest was lactic acid levels. In addition to describing this outcome as a continuous variable, we also generated binary lactate variables at two cut-offs, 3.5 and 5 mmol/L. The former was associated with an increased risk of severe outcomes among hospitalized children with pneumonia in Uganda, and the latter was associated with an increased risk of mortality among hospitalized febrile children in Tanzania.^17^,^25^ Secondary outcomes included the number of children with fast breathing, with positive mRDT results and those who received antibiotic treatment. The danger sign outcome was defined as presenting with ≥1 of the following symptoms: convulsions, coma, chest in-drawing, vomiting or not eating at the initial visit.^26^,^27^

### Statistical analysis

Data were double entered in the REDCap database to minimize the chance of error and analyzed with Stata 17.0 software (StataCorpsLLC, College Station, TX, USA). We summarized participant characteristics using descriptive statistics. Using Fisher's exact or χ^2^ testing, we examined the potential association between elevated lactate measurements and clinical characteristics and mRDT results at the initial visit.

## Results

### Study population

**Figure 2.**
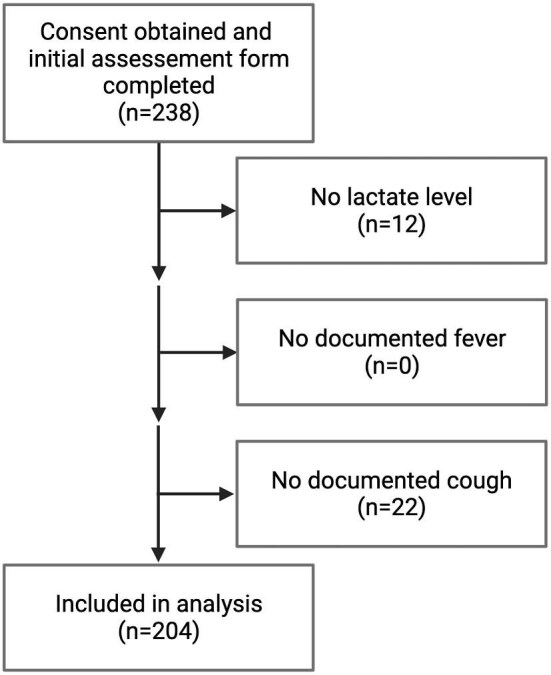
Flowchart of study enrollment and determination of study population for analysis. Created with BioRender.com.

During the study period, 238 children were enrolled and underwent an initial assessment by a study VHW (Figure 2). Twelve did not have a lactate measurement documented, and 22 did not have documented cough; these 34 (14.3%) children were excluded from the analysis.

### Participant demographics and clinical presentation

Demographic and clinical characteristics of study participants are shown in Tables 1 and 2, respectively. The median age of the 204 participants was 23 (quartile 1 [Q1]–quartile 3 [Q3]: 9–36) mo. The most common symptoms reported at the initial visit (other than fever and cough) were runny nose, headache and anorexia (i.e. poor feeding or not eating). Fast breathing was a reported symptom among 15 (7.4%) of participants. The proportion of participants whose caregivers reported ≥1 danger sign(s) at presentation was 15.2% (31/204). All of these participants either had vomiting and/or were not eating; none presented with coma, convulsions or chest in-drawing. The median duration of fever at time of presentation was 2 (Q1–Q3: 1–2) d.

**Table 1.** Demographic characteristics of the study participants. All values shown are n (%), except for age (in months) and family size (for which median and IQR are reported)

	Initial visit (n=204)
Variable	Frequency (%)
Sex	
- Male	91 (44.61)
- Female	113 (55.39)
Age (mo)	23 (9, 36)
Age ≤1 y^1^	87 (44.62)
Parish	
- Bugoye	6 (2.94)
- Katooke	67 (32.84)
- Muhambo	131 (64.22)
Occupation^2^	
- Businessman	1 (0.005)
- Midwife	1 (0.005)
- Peasant	191 (0.96)
- Tailor	1 (0.005)
- Teacher	4 (0.02)
Home construction^3^	
- Permanent	89 (45.41)
- Semipermanent	86 (43.88)
- Temporary	21 (10.71)
Family size^4^	6 (4, 8)

1 n=195.

2 n=198.

3 n=196.

4 n=187.

**Table 2.** Clinical characteristics of enrolled children at presentation. Results are reported as n (%), except for days since first fever and lactate measurements, which are reported as median and IQR

	Initial visit (n=204)	
Variable	Frequency (%)	
Presenting symptoms		
Runny nose	35 (17.2)	
Headache	30 (14.7)	
Not eating	20 (9.8)	
Diarrhea	16 (7.8)	
Fast breathing	15 (7.4)	
Vomiting	15 (7.4)	
Any danger sign	31 (15.2)	
Tachypnea^1^,^2^	25 33.4)	
Received malaria treatment in the last 2 wk^3^	2 (2.15)	
Received antibiotics in the last 2 wk^4^	6 (6.59)	
mRDT result^5^		
- Negative	139 (69.50)	
- Positive	61 (30.50)	
Antibiotics prescribed^6^	47 (23.38 )	
Lactate ≥5 mmol/L	9 (4.41)	
Referred to HCF	5 (2.45)	
	Median	Q1, Q3
Days since first fever^7^	2	(1,2)
Lactate (mmol/L)	2.1	(1.4, 2.9)

1 Defined as elevated respiratory rate for age: >50 breaths per minute (bpm) if age <12 mo, >40 bpm if age ≥1 y; n=74.

2 n=74

3 n=93.

4 n=91.

5 n=200.

6 n=201.

7 n=99.

### Initial assessment and management

**Figure 3.**
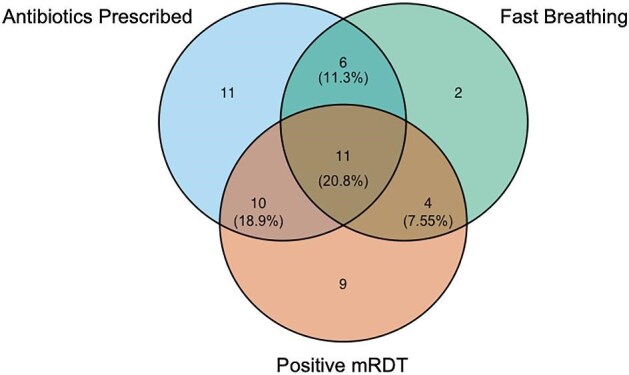
Venn diagram illustrating the frequency of key characteristics of the clinical presentation, evaluation and management among children presenting with fever and cough to the village health workers. A total of 72 children had data available for all three variables; the 53 who were positive for at least one of the three variables are shown.

Of the 74 participants for whom a respiratory rate (RR) was measured and documented by the VHW, 25 (33.7%) were tachypneic for age (Table 2). An elevated RR at initial assessment was associated with both treatment with antibiotics (p=0.018) and a positive mRDT result (p=0.036), but not with referral to a health facility. Of the 200 participants who underwent testing with an mRDT, 139 (69.5%) tested negative (Table 2). Of the 201 participants for whom antibiotic prescription data were available, 47 (23.4%) received antibiotics (Table 2), including 26 of the 61 individuals (42.6%) who tested positive for malaria by mRDT. Conversely, only 14.7% (20/136) of the mRDT-negative participants received antibiotics. The overlap between mRDT result, presence or absence of tachypnea, and whether or not antibiotics were prescribed at the initial visit, is shown in Figure 3. Of the 72 participants with data available for all three variables, 19 (26%) were mRDT negative with normal RR and did not receive antibiotics and therefore are not shown in Figure 3.

### Lactate testing

**Figure 4.**
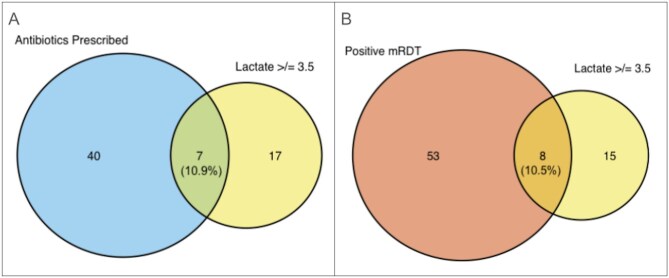
The proportion of participants with lactate ≥3.5 mmol/L and (A) antibiotic prescription at initial assessment and (B) positive mRDT.

The median lactate level was 2.1 (Q1–Q3: 1.4–2.9) mmol/L (Table 2). Only nine children, accounting for 4% of all participants, had a lactate level of ≥5 mmol/L (Table 3). Three of these children (33%) had a positive mRDT (for comparison, 31% of the full cohort was mRDT-positive), and only two reported any danger signs. Five of the nine (55.6%) were referred to a healthcare facility as per the iCCM and study protocols. Of those with lactate ≥5 mmol/L, only two (22%) received antibiotics from the VHWs. Twenty-four participants (12%) had a lactate level of ≥3.5 mmol/L; the proportion of participants who were aged ≤1 y did not differ between those that had a lactate level above or below 3.5 mmol/L (data not shown). Of the 24 with lactate ≥3.5 mmol/L, seven (29%) received antibiotics (Figure 4).

Of the 61 participants with a positive mRDT, eight (13%) had a lactate ≥3.5 mmol/L (Figure 4) and three (5%) had a lactate ≥5 mmol/L. The proportion of participants who presented with ≥1 danger sign(s), who were mRDT positive, had fast breathing or received antibiotics during the study follow-up, did not differ between those with lactate ≥5 and <5 mmol/L or between those with lactate of ≥3.5 or <3.5 mmol/L (Table 4).

**Table 3.** Demographic, clinical and outcome information for children with a lactate measurement of ≥5 mmol/L

Age (mo)	Sex	mRDT result	Danger signs reported	Lactate (mmol/L)	Antibiotic treatment given	Disposition
48	Female	Negative	None	6.0	Yes	Home
6	Female	Positive	None	6.1	Yes	Home
37	Male	Negative	None	5.0	No	Referred to HF
5	Male	Negative	None	8.5	No	Referred to HF
27	Female	Positive	None	5.2	No	Home
8	Female	Negative	Fast breathing	6.1	No	Referred to HF
48	Male	Negative	None	6.5	No	Home
48	Male	Positive	Not eating	6.0	No	Referred to HF
4	Female	Negative	None	5.8	No	Referred to HF

HF: health facility; mRDT: malaria rapid diagnostic test.

**Table 4.** Association between elevated lactate levels and severity of presentation and negative clinical outcomes. p-values are from Fisher exact tests, except where indicated

	Lactate ≥3.5 mmol/L	Lactate ≥5 mmol/L
Variable	p-value	p-value
mRDT-positive	0.635*	1.000
Danger signs	0.543	1.000
Tachypnea	0.682	0.262
Antibiotics prescribed	0.476^#^	1.000

* χ^2^=0.225.

# χ^2^=0.510.

### Referrals

Of the 204 children who presented for an initial visit to the VHW, five (2.5%) were referred to a health facility, all of whom had lactate levels of ≥5 mmol/L. Three of the five were aged <1 y and one was mRDT-positive. None were prescribed antibiotics at the initial visit.

## Discussion

In this study, VHWs in southwestern Uganda used point-of-care meters to measure lactate levels among children aged <5 y presenting with fever and cough. Overall, participants generally had low lactic acid levels and only five of the 204 children were referred to a health facility, all of whom had lactate levels ≥5 mmol/L. The remaining four children with lactate levels of ≥5 mmol/L did not have physical manifestations that led to recognition of danger signs and, in turn, referral. Although we did not find that elevated lactate was associated with correlates of illness severity at presentation, given that elevated lactate has been associated with adverse outcomes in many other contexts and studies,^21^ this is a concerning finding. This emphasizes the potential value of the addition of a point-of-care lactate measurement to the VHWs’ protocols for identification of children who could be at high risk of poor clinical outcomes.

The overall low lactate levels that we noted are likely partially explained by presentation to the VHWs early in illness, with caregivers in our cohort reporting a median of 2 d of fever prior to presentation to the VHW. Recent studies in Uganda and other countries in SSA have shown improved health-seeking behavior from community members and quality of care in both rural and urban settings with VHWs.^23^,^28^ In general, caregivers in our setting have positive interactions with, and trust of VHWs, as a capable team to support in disease detection, treatment and/or referral, and have sought early treatment for the conditions of malaria, diarrhea and pneumonia for many years now; the pattern of care-seeking early in illness that we observed supports this. We also noted low lactate levels, even in the subgroup of children who were mRDT-positive, in contrast to previous literature.^29^ However, in our previous study of children presenting to a health facility with febrile ARI, and therefore perhaps more likely to be sicker than those presenting to VHWs, lactate levels were also overall low.^30^ Therefore, our findings may simply reflect that the majority of children with febrile ARI in this region, regardless of where they first seek care, have mild illness.

Elevated lactate levels were not associated with danger signs, tachypnea or antibiotic use at presentation (correlates of more severe illness). Although this is inconsistent with past research, in which elevated lactate levels were associated with severity of febrile illness and mortality among children hospitalized with pneumonia, our broad definition of danger signs may partly explain this finding. For instance, we likely included children with mild vomiting and decreased appetite, rather than strictly those experiencing more severe symptoms, such as vomiting everything and being unable to breastfeed or drink, as the danger signs are defined in the iCCM sick child job aid for iCCM.^31^

Referral practices among VHWs and patient adherence to the referral recommendation when given are highly variable in southwestern Uganda.^14^,^32^ In our study, four of the nine children with lactate levels ≥5 mmol/L were not referred to the health facility as per the study protocol. In addition, the iCCM guidelines recommend referral of mRDT-negative fever cases to the health facility; in our cohort, 135 of 139 mRDT-negative children were not referred. There are known factors, such as long distance to the nearest preferred health facility and the need to care for other children, that hinder referral in this region; it is possible that this may influence a VHW's recommendation.^11^,^31^,^32^ However, we also observed that 21 children were prescribed antibiotics despite not being tachypneic for age, including 10 children who were mRDT-positive. Therefore, despite the training received by the VHW through the study and as part of the iCCM program, our findings suggest adherence to guidelines may be inconsistent. Continuous refresher training and support supervision have been shown to improve the performance of VHWs in numerous studies.^33^

Our study has several strengths. To the best of our knowledge, this is the first study to implement lactate testing at the point-of-care in routine iCCM. In addition, our study population is likely a representative sample of children who present to VHWs in the region. However, the study also has limitations. First, after completing initial training, VHWs were not directly supervised when using the lactate meter. This raises the possibility of user error. However, the VHWs demonstrated proficiency in training and have shown the ability to maintain skills with tools such as mRDT.^23^,^34^ Second, the primary outcome—lactate levels ≥5 mmol/L—occurred in only a small proportion of participants. This limited our ability to estimate the potential association of lactate measurement with clinical presentation. Lastly, the study was initially designed with a follow-up visit to record the clinical outcomes following either prescription or referral away from the VHW’s home, but as discussed in the Methods section, these assessments were not consistently completed, and therefore there was a significant amount of missing data that prevented us from being able to report and draw conclusions from the follow-up visits. This was most likely a training gap that should be mitigated in future studies as understanding the relationship between lactate levels and clinical outcomes is key to determining if and how point-of-care measurement would be useful for improving risk stratification of ill children in the community.

## Conclusions

Integrating point-of-care lactate testing into the workflows of iCCM programs appears feasible. Lactic acidosis was not associated with illness severity at presentation; however, very few children in our cohort had elevated lactate levels, which likely reflects that children presenting to VHWs often have mild symptoms or are early in illness. Routine physical examination failed to identify nearly one-half of children with elevated lactate levels, which suggests that simple, convenient tools may play an important role in risk stratification. Our findings provide a rationale for larger studies focused on clinical outcomes to determine the utility and cost-effectiveness of routine lactate measurement at the community health worker level.

## Data Availability

De-identified individual data that support the results will be shared following publication upon request provided the investigator who proposes to use the data has approval from an Institutional Review Board (IRB), Independent Ethics Committee (IEC) or Research Ethics Board (REB), as applicable, and executes a data use/sharing agreement with UNC. Researchers may apply for data access by contacting the corresponding author.
